# Bis[4-(dimethyl­amino)phen­yl]diazene oxide

**DOI:** 10.1107/S1600536808006740

**Published:** 2008-04-18

**Authors:** Graeme J. Gainsford, M. Delower H. Bhuiyan, Andrew J. Kay

**Affiliations:** a Industrial Research Limited, PO Box 31-310, Lower Hutt, New Zealand

## Abstract

The asymmetric unit of the title compound, C_16_H_20_N_4_O, contains six independent approximately planar mol­ecules and is best described as a commensurate modulation of a *P*2_1_/*c* parent. Two sets of disordered mol­ecules share almost the same locations (related by an in-plane translation), ensuring that the *c*-glide plane condition is not attained. C—H⋯O inter­actions provide structural cohesion. The site occupancy factors of the disordered molecules are *ca* 0.72/0.28 and 0.67/0.33.

## Related literature

For general background, see: Kay *et al.* (2004 ▶); Gainsford *et al.* (2007 ▶, 2008 ▶). For related structures, see: Greci *et al.* (2003 ▶); Domański *et al.* (2001 ▶); Ejsmont *et al.* (2002 ▶); Browning *et al.* (1974 ▶). For related literature, see: Allen (2002 ▶); Desiraju & Steiner (1999 ▶); Flack (1983 ▶); Flack & Bernardinelli (2000 ▶).
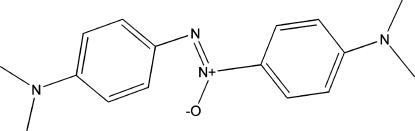


## Experimental

### 

#### Crystal data

C_16_H_20_N_4_O
*M* *_r_* = 284.36
Monoclinic, 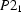

*a* = 12.0080 (6) Å
*b* = 22.0379 (11) Å
*c* = 17.3408 (8) Å
β = 109.450 (3)°
*V* = 4327.0 (4) Å^3^
*Z* = 12
Mo *K*α radiation
μ = 0.09 mm^−1^
*T* = 99 (2) K
0.66 × 0.25 × 0.17 mm

#### Data collection

Bruker–Nonius APEX2 CCD area-detector diffractometer
Absorption correction: multi-scan (*SADABS*; Bruker, 2006 ▶; Blessing, 1995 ▶) *T*_min_ = 0.800, *T*_max_ = 1.000 (expected range = 0.789–0.986)
106040 measured reflections
13444 independent reflections
8097 reflections with *I* > 2σ(*I*)
*R*_int_ = 0.062

#### Refinement

*R*[*F* ^2^ > 2σ(*F* ^2^)] = 0.109
*wR*(*F* ^2^) = 0.374
*S* = 1.03
13444 reflections
1113 parameters
1 restraint
H-atom parameters constrained
Δρ_max_ = 0.79 e Å^−3^
Δρ_min_ = −0.69 e Å^−3^

### 

Data collection: *APEX2* (Bruker, 2006 ▶); cell refinement: *SAINT* (Bruker, 2006 ▶); data reduction: *SAINT* and *SADABS* (Bruker, 2006 ▶); program(s) used to solve structure: *SHELXS97* (Sheldrick, 2008 ▶); program(s) used to refine structure: *SHELXL97* (Sheldrick, 2008 ▶); molecular graphics: *ORTEP-3* (Farrugia, 1997 ▶), *PLATON* (Spek, 2003 ▶) and *Mercury* (Macrae *et al.*, 2006 ▶); software used to prepare material for publication: *SHELXL97*, *PLATON* and *Mercury*.

## Supplementary Material

Crystal structure: contains datablocks global, I. DOI: 10.1107/S1600536808006740/sj2473sup1.cif

Structure factors: contains datablocks I. DOI: 10.1107/S1600536808006740/sj2473Isup2.hkl

Additional supplementary materials:  crystallographic information; 3D view; checkCIF report

## Figures and Tables

**Table 1:** Hydrogen-bond geometry (Å, °)

*D*—H⋯*A*	*D*—H	H⋯*A*	*D*⋯*A*	*D*—H⋯*A*
C15—H15⋯O11	0.95	2.35	2.708 (8)	101
C25—H25⋯O21	0.95	2.41	2.745 (8)	101
C35—H35⋯O31	0.95	2.32	2.651 (12)	100
C45—H45⋯O41	0.95	2.36	2.687 (12)	100
C55—H55⋯O51	0.95	2.38	2.722 (12)	100
C65—H65⋯O61	0.95	2.33	2.663 (16)	100
C110—H110⋯O11	0.95	2.15	2.742 (8)	119
C114—H114⋯O61^i^	0.95	2.51	3.328 (12)	144
C210—H210⋯O21	0.95	2.13	2.727 (10)	120
C214—H214⋯O31^ii^	0.95	2.50	3.402 (10)	158
C310—H310⋯O31	0.95	2.21	2.750 (12)	115
C410—H410⋯O41	0.95	2.17	2.732 (11)	117
C414—H414⋯O51	0.95	2.57	3.448 (12)	153
C510—H510⋯O51	0.95	2.09	2.684 (11)	120
C514—H514⋯O41^i^	0.95	2.48	3.323 (11)	148
C610—H610⋯O61	0.95	2.20	2.726 (16)	115
C614—H614⋯O11	0.95	2.58	3.471 (12)	156

Symmetry codes: (i) 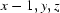; (ii) 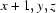.

**Fig. 1.**
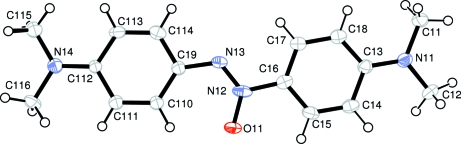
Molecular structure of molecule 1 of the asymmetic unit (Farrugia, 1997); displacement ellipsoids are shown at the 50% probability level.

**Fig. 2.**
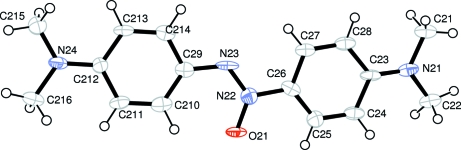
Molecular structure of molecule 2 of the asymmetic unit (Farrugia, 1997); displacement ellipsoids are shown at the 50% probability level.

**Fig. 3.**
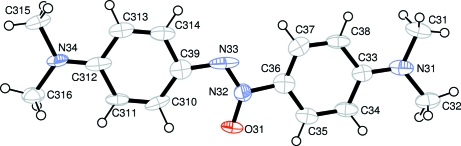
Molecular structure of molecule 3 of the asymmetic unit (Farrugia, 1997); displacement ellipsoids are shown at the 50% probability level.

**Fig. 4.**
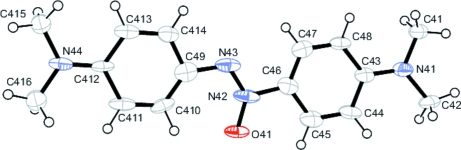
Molecular structure of molecule 4 of the asymmetic unit (Farrugia, 1997); displacement ellipsoids are shown at the 50% probability level.

**Fig. 5.**
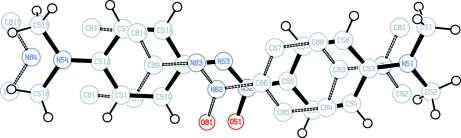
PLATON (Spek, 2003) view of the disordered molecular unit based on molecule 5 (see text). Occupancies of the O51/O81 based units are 0.724 (6)/0.276 (6).

**Fig. 6.**
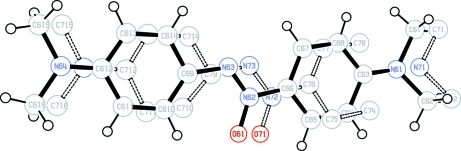
PLATON (Spek, 2003) view of the disordered molecular unit based on molecule 6 (see text). Occupancies of the O61/O71 based units are 0.674 (7)/0.326 (7).

**Fig. 7.**
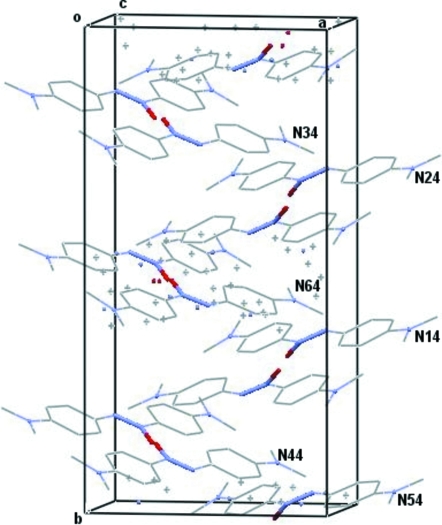
Packing diagram (Mercury; Macrae et al., 2006) of the unit cell showing the 1/3 relationship down the b axis. One terminal methylamino (Nx4) of each unique molecule is labelled. Minor disorder molecules breaking c glide symmetry are shown as "+" signs. Hydrogen atoms are omitted for clarity.

## supplementary crystallographic information

### Comment

During the preparation of the chromophore 2-{3-cyano-4-[(4-dimethylamino-phenylimino)-methyl]-5,5-dimethyl-5H -furan-2-ylidene}-malonitrile we isolated the title compound as a byproduct. There are at least 27 reported structures containing a 4-azoxybenzene moiety (Cambridge Structural Database, Version 5.29 with November 2007 updates; Allen, 2002). Of these, four are closely related or have been extensively analysed: 1-(4-Aminophenyl)-2-phenyldiazene 2-oxide (α & β) which crystallized as different polymorphs (QIKFEV, Domański *et al.*(2001); QIKFEV01, Ejsmont *et al.* (2002)), the *p*-dimethylaminoazoxybenzene (MAZXBZ, Browning *et al.* (1974)) and the 3-chloro adduct which was also isolated as a side product (EMUQOS, Greci *et al.*, 2003).

The asymmetric unit contains six copies of the title *chiro* compound which are shown in Figures 1–6. The structure can be described as a commensurate modulation of a *P*2_1_/*c* parent with 1/3 the *b* axis (Figure 7). Figures 5 & 6 depict the disordered molecules 5 & 6, with the minor components (given numbers 8 & 7 respectively) related by a translation along the molecule by 1–1.5Å (*i.e.* close to a bond length): hence in Figure 6, atoms N61 & C73 coincide and were refined on one site (see experimental). The convention we used for atom labelling is C(N,*O*)xn where *x* is the molecule number and n is the atom number (see Tables 1 & 2). Because of the approximations involved in refining the two disordered molecules (5,8 & 6,7) (reflected in larger su values), we use only average dimensions for molecules 1–4 in the discussion below. It is however noted that all the structures (1–6) are nearly superimposable with weighted r.m.s. fits ranging from 0.016Å to 0.071Å (Spek, 2003).

Overall the molecules are close to being planar with average interplanar angles between the two phenyl rings of 3.4 (3)°. From the previous studies of the *trans*-4-aminoazoxybenzenes (QIKFEV, QIKFEV01) it is clear that the molecules found here are similar to the QIKFEV α isomer with the average C_aryl_—NO length longer than the C_aryl_—N (1.474 *versus* 1.456 Å). The dimethylamino nitrogen atoms (Nx1 & Nx4) are close to planar with their bound atoms (Cx1,Cx2,Cx3 & Cx12,Cx15,Cx16): average out of plane deviations are 0.019 (8) and 0.035 (9)Å respectively similar to EMUQOS values. The (average) dimensions of the two phenyl rings do not support any quinonoid deformation. The character of the substituent to the phenyl rings (quoted by Domanski *et al.* (2001)) being electron-releasing is observed with the *ipso* angles at Cx3, Cx12 & Cx9 being 118.0 (4), 117.6 (4) & 117.6 (5)° respectively compared with that at Cx6 (120.4 (1)°). In the studies noted, there is some ambivalence about the intramolecular contact between Ox1 and Hx10(on Cx10) as to whether it is attractive or not. The average Hx5···Ox1 and Cx5–Hx5···Ox1 dimensions (2.36 Å) and angle (100°) compare with the Hx10···Ox1 values of 2.16Å and 118°, confirming the latter are attractive weak interactions (Desiraju & Steiner (1999)).

The Cx14—Hx14···O(*n*)1 intermolecular interactions, involving the proton on the opposite side of the Cx9—Cx14 ring to the intramolecular interaction, links non-parallel molecules into dimers (Table 2), as has been observed before in MAZXBZ with H···O 2.44Å and C—H···O 172°. The resulting "herringbone" structure has molecules in approximate layers parallel to the (1,2,-1) and (-1,2,1) planes.

### Experimental

To a solution of 4,5,5-trimethyl-3-cyano-2(5*H*)-furanylidenepropane dinitrile (1.0 g, 5.02 mmol) in 20 ml of absolute ethanol was added the *N*,*N*-dimethylamino-4-nitrosoaniline (829 mg, 5.52 mmol) and the reaction mixture was refluxed for 1 h. in the presence of piperidine acetate as catalyst. This reaction mixture was then cooled and filtered. The crude material was chromatographed (silica gel, dichloromethane) to afford 4,4'-bis(dimethylamino)azoxybenzene (71 mg, 5%), (I), as a brownish red solid. This was the third major product eluted from the column. m.p. 250–252°C; ^1^H NMR (300 MHz, DMSO) δ 3.02 (12 H, s, 4 *x* CH_3_), 6.77 (4 H, d, *J* 12.0 Hz, ArH), 8.03(2 H, d, *J* 9.0 Hz, ArH), 8.16 (2 H, d, *J* 9.0 Hz, ArH),; ^13^C NMR (75 MHz, DMSO) δ 39.2, 111.2, 111.4, 122.9, 127.6. *M*+ *m/z* for C_16_H_21_N_4_O 285.1715, Found 285.1704.

### Refinement

In the absence of significant anomalous scattering, the values of the Flack (1983) parameter were indeterminate (Flack & Bernardinelli, 2000). Accordingly, the Friedel-equivalent reflections were merged prior to the final refinements. A total of 16 reflections were omitted from the refinement; all but 3 were measured at low theta with unbalanced backgrounds resulting in (outlier) negative intensities.

Preliminary analysis (structural solution) indicated that the space group might be *P*2_1_/*c* (at the 94% level (Spek, 2003)) but this was ruled out by the definite breaking of the c glide absence condition. The central N=N+(O–) is often disordered in these structures; the final difference Fourier maps indicate that this may be partially the case for molecules 3 & 4. Refinement was only successful in the reported space group. The disordered fragments based on O71 and O81 (corresponding to molecules O61 & O51 respectively, see Figures 5 & 7) were each given one common *U*_iso_ value and a complementary occupancy to the adjacent one-bond shifted molecule: so molecules 6/7 had occupancies of 0.674 (7)/0.326 (7) with *U*_iso_ 0.0392 (16) Å^2^ (molecule 7) and 5/8 occupancies of 0.724 (6)/0.276 (6) with *U*_iso_ 0.0332 (15) Å^2^ (molecule 8). H atoms on the minor disordered molecules (O71 & O81) were not located or included.

Atoms that were unstable to refinement as anisotropic atoms, or gave highly prolate parameters, were refined with isotropic thermal parameters.

All H atoms bound to carbon were constrained to their expected geometries (C—H 0.98, 0.99 Å). All methyl and tertiary H atoms were refined with *U*_iso_ 1.5 & 1.2 times respectively that of the *U*_eq_ of their parent atom.

### Crystal data

**Table:**

C_16_H_20_N_4_O	*F*_000_ = 1824
*M**_r_* = 284.36	*D*_x_ = 1.310 Mg m^−^^3^
Monoclinic, *P*2_1_	Mo *K*α radiation λ = 0.71073 Å
Hall symbol: P 2yb	Cell parameters from 8860 reflections
*a* = 12.0080 (6) Å	θ = 2.5–29.5º
*b* = 22.0379 (11) Å	µ = 0.09 mm^−^^1^
*c* = 17.3408 (8) Å	*T* = 99 (2) K
β = 109.450 (3)º	Needles, orange
*V* = 4327.0 (4) Å^3^	0.66 × 0.25 × 0.17 mm
*Z* = 12	

### Data collection

**Table:**

Bruker–Nonius APEX2 CCD area-detector diffractometer	13444 independent reflections
Radiation source: fine-focus sealed tube	8097 reflections with *I* > 2σ(*I*)
Monochromator: graphite	*R*_int_ = 0.062
Detector resolution: 8.192 pixels mm^-1^	θ_max_ = 30.6º
*T* = 99(2) K	θ_min_ = 1.8º
φ and ω scans	*h* = −17→17
Absorption correction: multi-scan(SADABS; Bruker, 2006; Blessing, 1995)	*k* = −31→31
*T*_min_ = 0.800, *T*_max_ = 1.0	*l* = −24→24
106040 measured reflections	

### Refinement

**Table:**

Refinement on *F*^2^	Secondary atom site location: difference Fourier map
Least-squares matrix: full	Hydrogen site location: inferred from neighbouring sites
*R*[*F*^2^ > 2σ(*F*^2^)] = 0.109	H-atom parameters constrained
*wR*(*F*^2^) = 0.374	*w* = 1/[σ^2^(*F*_o_^2^) + (0.167*P*)^2^ + 10.2925*P*] where *P* = (*F*_o_^2^ + 2*F*_c_^2^)/3
*S* = 1.03	(Δ/σ)_max_ = 0.050
13444 reflections	Δρ_max_ = 0.79 e Å^−^^3^
1113 parameters	Δρ_min_ = −0.69 e Å^−^^3^
1 restraint	Extinction correction: none
Primary atom site location: structure-invariant direct methods	

### Special details

**Table:**

Geometry. All e.s.d.'s (except the e.s.d. in the dihedral angle between two l.s. planes) are estimated using the full covariance matrix. The cell e.s.d.'s are taken into account individually in the estimation of e.s.d.'s in distances, angles and torsion angles; correlations between e.s.d.'s in cell parameters are only used when they are defined by crystal symmetry. An approximate (isotropic) treatment of cell e.s.d.'s is used for estimating e.s.d.'s involving l.s. planes.
Refinement. Refinement of *F*^2^ against ALL reflections. The weighted *R*-factor *wR* and goodness of fit *S* are based on *F*^2^, conventional *R*-factors *R* are based on *F*, with *F* set to zero for negative *F*^2^. The threshold expression of *F*^2^ > σ(*F*^2^) is used only for calculating *R*-factors(gt) *etc*. and is not relevant to the choice of reflections for refinement. *R*-factors based on *F*^2^ are statistically about twice as large as those based on *F*, and *R*- factors based on ALL data will be even larger.

### Fractional atomic coordinates and isotropic or equivalent isotropic displacement parameters (Å^2^)

**Table:**

	*x*	*y*	*z*	*U*_iso_*/*U*_eq_	Occ. (<1)
O11	0.2603 (4)	0.1917 (2)	0.2315 (3)	0.0314 (11)	
N11	0.4478 (5)	0.1429 (3)	0.6120 (4)	0.0285 (12)	
N12	0.2049 (5)	0.1611 (3)	0.2714 (4)	0.0296 (12)	
N13	0.1027 (5)	0.1379 (2)	0.2445 (3)	0.0238 (10)	
N14	−0.2037 (5)	0.1412 (3)	−0.0806 (3)	0.0299 (12)*	
C11	0.4004 (7)	0.1082 (4)	0.6655 (5)	0.0342 (15)	
H11A	0.3248	0.1257	0.6641	0.051*	
H11B	0.4562	0.1096	0.7216	0.051*	
H11C	0.3886	0.0660	0.6469	0.051*	
C12	0.5543 (6)	0.1753 (4)	0.6473 (5)	0.0371 (16)	
H12A	0.6204	0.1516	0.6420	0.056*	
H12B	0.5666	0.1826	0.7054	0.056*	
H12C	0.5497	0.2142	0.6191	0.056*	
C13	0.3877 (5)	0.1446 (3)	0.5288 (4)	0.0273 (13)	
C14	0.4370 (6)	0.1769 (3)	0.4765 (4)	0.0272 (13)	
H14	0.5116	0.1964	0.4985	0.033*	
C15	0.3754 (6)	0.1797 (3)	0.3941 (4)	0.0306 (14)	
H15	0.4098	0.2006	0.3596	0.037*	
C16	0.2679 (6)	0.1539 (3)	0.3598 (4)	0.0243 (12)	
C17	0.2170 (6)	0.1222 (3)	0.4099 (4)	0.0266 (13)	
H17	0.1415	0.1040	0.3866	0.032*	
C18	0.2767 (6)	0.1174 (3)	0.4934 (4)	0.0271 (13)	
H18	0.2421	0.0954	0.5268	0.032*	
C19	0.0346 (6)	0.1450 (3)	0.1614 (4)	0.0243 (12)*	
C110	0.0650 (6)	0.1720 (3)	0.0964 (4)	0.0256 (12)*	
H110	0.1412	0.1892	0.1073	0.031*	
C111	−0.0159 (6)	0.1735 (3)	0.0168 (4)	0.0266 (12)*	
H111	0.0031	0.1946	−0.0250	0.032*	
C112	−0.1269 (5)	0.1432 (3)	−0.0017 (3)	0.0219 (11)*	
C113	−0.1568 (6)	0.1185 (3)	0.0639 (4)	0.0278 (13)	
H113	−0.2339	0.1025	0.0539	0.033*	
C114	−0.0770 (6)	0.1169 (3)	0.1416 (4)	0.0282 (13)	
H114	−0.0973	0.0964	0.1832	0.034*	
C115	−0.3180 (6)	0.1151 (3)	−0.1004 (4)	0.0325 (14)*	
H15A	−0.3136	0.0783	−0.0678	0.049*	
H15B	−0.3477	0.1048	−0.1587	0.049*	
H15C	−0.3716	0.1443	−0.0884	0.049*	
C116	−0.1732 (7)	0.1686 (4)	−0.1472 (4)	0.0343 (15)	
H16A	−0.1949	0.2116	−0.1516	0.052*	
H16B	−0.2160	0.1479	−0.1985	0.052*	
H16C	−0.0880	0.1647	−0.1363	0.052*	
O21	0.7507 (4)	0.3591 (3)	0.7787 (3)	0.0356 (12)	
N21	0.5621 (5)	0.3088 (3)	0.3987 (4)	0.0354 (13)	
N22	0.8010 (5)	0.3283 (3)	0.7351 (4)	0.0339 (13)*	
N23	0.9052 (5)	0.3045 (3)	0.7629 (4)	0.0314 (13)	
N24	1.2173 (5)	0.3106 (3)	1.0890 (4)	0.0342 (13)	
C21	0.6101 (7)	0.2753 (4)	0.3452 (5)	0.0402 (17)	
H21A	0.5556	0.2779	0.2889	0.060*	
H21B	0.6206	0.2327	0.3623	0.060*	
H21C	0.6866	0.2926	0.3482	0.060*	
C22	0.4564 (7)	0.3421 (4)	0.3643 (6)	0.0438 (19)	
H22A	0.3942	0.3245	0.3822	0.066*	
H22B	0.4324	0.3404	0.3045	0.066*	
H22C	0.4695	0.3844	0.3823	0.066*	
C23	0.6200 (6)	0.3111 (3)	0.4801 (5)	0.0299 (14)	
C24	0.5710 (7)	0.3433 (3)	0.5320 (5)	0.0340 (15)	
H24	0.4966	0.3628	0.5095	0.041*	
C25	0.6305 (6)	0.3464 (4)	0.6145 (4)	0.0349 (15)*	
H25	0.5947	0.3662	0.6489	0.042*	
C26	0.7402 (7)	0.3217 (3)	0.6480 (4)	0.0286 (13)	
C27	0.7936 (7)	0.2906 (3)	0.5994 (5)	0.0324 (14)	
H27	0.8692	0.2726	0.6234	0.039*	
C28	0.7353 (6)	0.2863 (3)	0.5159 (5)	0.0327 (15)	
H28	0.7726	0.2667	0.4823	0.039*	
C29	0.9751 (6)	0.3115 (3)	0.8476 (4)	0.0300 (13)	
C210	0.9447 (6)	0.3369 (3)	0.9122 (5)	0.0343 (16)	
H210	0.8674	0.3526	0.9016	0.041*	
C211	1.0259 (6)	0.3395 (3)	0.9919 (5)	0.0321 (14)	
H211	1.0066	0.3609	1.0333	0.039*	
C212	1.1371 (6)	0.3100 (3)	1.0105 (4)	0.0278 (13)*	
C214	1.0861 (7)	0.2834 (3)	0.8665 (5)	0.0321 (14)	
H214	1.1060	0.2629	0.8246	0.039*	
C213	1.1669 (6)	0.2850 (3)	0.9447 (4)	0.0301 (14)	
H213	1.2439	0.2690	0.9546	0.036*	
C215	1.3306 (7)	0.2831 (4)	1.1090 (5)	0.0378 (16)*	
H25A	1.3229	0.2434	1.0822	0.057*	
H25B	1.3644	0.2779	1.1684	0.057*	
H25C	1.3825	0.3091	1.0901	0.057*	
C216	1.1852 (8)	0.3344 (4)	1.1571 (5)	0.0416 (18)	
H26A	1.2002	0.3782	1.1619	0.062*	
H26B	1.2327	0.3143	1.2078	0.062*	
H26C	1.1013	0.3268	1.1474	0.062*	
O31	0.2305 (5)	0.2080 (3)	0.7569 (4)	0.0414 (13)	
N31	0.0438 (7)	0.2581 (4)	0.3779 (6)	0.052 (2)	
N32	0.2833 (6)	0.2369 (3)	0.7173 (4)	0.0394 (14)*	
N33	0.3846 (8)	0.2583 (3)	0.7408 (6)	0.056 (2)	
N34	0.6924 (5)	0.2553 (3)	1.0709 (4)	0.0403 (15)	
C31	0.0907 (9)	0.2913 (5)	0.3239 (6)	0.052 (2)	
H31A	0.0954	0.3345	0.3380	0.078*	
H31B	0.0388	0.2859	0.2672	0.078*	
H31C	0.1698	0.2761	0.3297	0.078*	
C32	−0.0663 (8)	0.2233 (5)	0.3395 (7)	0.054 (2)	
H32A	−0.1269	0.2367	0.3621	0.081*	
H32B	−0.0508	0.1799	0.3506	0.081*	
H32C	−0.0942	0.2301	0.2803	0.081*	
C33	0.1018 (7)	0.2551 (4)	0.4614 (5)	0.0385 (17)	
C34	0.0536 (7)	0.2251 (4)	0.5129 (6)	0.0424 (19)	
H34	−0.0223	0.2072	0.4901	0.051*	
C35	0.1127 (7)	0.2199 (4)	0.5985 (6)	0.0414 (18)	
H35	0.0781	0.1995	0.6332	0.050*	
C36	0.2245 (8)	0.2465 (4)	0.6288 (6)	0.046 (2)	
C37	0.2731 (8)	0.2789 (4)	0.5789 (6)	0.044 (2)	
H37	0.3481	0.2977	0.6017	0.053*	
C38	0.2120 (7)	0.2837 (4)	0.4962 (6)	0.0413 (19)	
H38	0.2447	0.3065	0.4625	0.050*	
C39	0.4524 (8)	0.2500 (4)	0.8300 (6)	0.0413 (18)	
C310	0.4268 (7)	0.2212 (3)	0.8940 (6)	0.0406 (19)	
H310	0.3542	0.2001	0.8833	0.049*	
C311	0.5055 (7)	0.2233 (3)	0.9720 (5)	0.0372 (17)	
H311	0.4840	0.2053	1.0148	0.045*	
C312	0.6153 (7)	0.2506 (3)	0.9911 (6)	0.044 (2)	
C313	0.6446 (7)	0.2772 (3)	0.9275 (6)	0.0403 (19)	
H313	0.7191	0.2963	0.9382	0.048*	
C314	0.5644 (8)	0.2757 (4)	0.8484 (6)	0.045 (2)	
H314	0.5866	0.2927	0.8054	0.054*	
C315	0.8103 (7)	0.2825 (4)	1.0885 (6)	0.049 (2)	
H35A	0.8483	0.2653	1.0514	0.074*	
H35B	0.8585	0.2737	1.1452	0.074*	
H35C	0.8024	0.3265	1.0806	0.074*	
C316	0.6622 (8)	0.2286 (5)	1.1377 (6)	0.050 (2)	
H36A	0.5973	0.2515	1.1461	0.074*	
H36B	0.7312	0.2298	1.1877	0.074*	
H36C	0.6378	0.1864	1.1244	0.074*	
O41	0.7975 (5)	0.3742 (3)	0.2746 (4)	0.0451 (14)	
N41	0.9841 (6)	0.4259 (4)	0.6541 (4)	0.0423 (15)	
N42	0.7443 (7)	0.4037 (3)	0.3130 (5)	0.0460 (18)	
N43	0.6439 (7)	0.4265 (3)	0.2899 (5)	0.0485 (18)	
N44	0.3356 (6)	0.4182 (3)	−0.0370 (5)	0.0414 (16)	
C41	0.9366 (7)	0.4595 (4)	0.7077 (5)	0.0397 (17)	
H41A	0.9390	0.5030	0.6968	0.060*	
H41B	0.9839	0.4512	0.7648	0.060*	
H41C	0.8547	0.4470	0.6980	0.060*	
C42	1.0936 (7)	0.3916 (4)	0.6925 (6)	0.0438 (19)	
H42A	1.1146	0.3935	0.7521	0.066*	
H42B	1.1574	0.4093	0.6763	0.066*	
H42C	1.0818	0.3492	0.6746	0.066*	
C43	0.9267 (6)	0.4240 (3)	0.5705 (5)	0.0342 (15)	
C44	0.9742 (7)	0.3915 (4)	0.5200 (6)	0.0431 (19)	
H44	1.0474	0.3712	0.5436	0.052*	
C45	0.9170 (8)	0.3880 (4)	0.4349 (6)	0.047 (2)	
H45	0.9535	0.3691	0.4002	0.056*	
C46	0.8027 (7)	0.4138 (4)	0.4032 (5)	0.0397 (17)	
C47	0.7565 (7)	0.4461 (4)	0.4530 (6)	0.0426 (18)	
H47	0.6826	0.4659	0.4297	0.051*	
C48	0.8136 (6)	0.4505 (3)	0.5349 (5)	0.0354 (15)	
H48	0.7776	0.4714	0.5683	0.043*	
C49	0.5769 (8)	0.4172 (4)	0.2026 (6)	0.0436 (19)	
C410	0.6031 (6)	0.3912 (4)	0.1390 (6)	0.0401 (18)	
H410	0.6795	0.3743	0.1495	0.048*	
C411	0.5249 (7)	0.3885 (3)	0.0613 (6)	0.0409 (19)	
H411	0.5446	0.3667	0.0204	0.049*	
C412	0.4141 (6)	0.4183 (3)	0.0416 (5)	0.0343 (15)	
C413	0.3850 (7)	0.4432 (3)	0.1062 (5)	0.0381 (18)	
H413	0.3080	0.4592	0.0961	0.046*	
C414	0.4664 (7)	0.4451 (4)	0.1855 (5)	0.0393 (16)	
H414	0.4469	0.4653	0.2277	0.047*	
C415	0.2199 (7)	0.4465 (4)	−0.0554 (5)	0.0433 (17)*	
H45A	0.1646	0.4280	−0.1047	0.065*	
H45B	0.2264	0.4900	−0.0644	0.065*	
H45C	0.1910	0.4404	−0.0093	0.065*	
C416	0.3707 (8)	0.3947 (4)	−0.1030 (5)	0.0470 (19)*	
H46A	0.4255	0.4232	−0.1150	0.071*	
H46B	0.3008	0.3896	−0.1518	0.071*	
H46C	0.4098	0.3554	−0.0872	0.071*	
O51	0.3067 (6)	0.5245 (4)	0.2846 (4)	0.0333 (15)	0.724 (6)
N51	0.4915 (6)	0.4732 (4)	0.6635 (5)	0.0276 (15)	0.724 (6)
N52	0.2534 (6)	0.4933 (3)	0.3272 (5)	0.0223 (15)*	0.724 (6)
N53	0.1494 (6)	0.4704 (4)	0.2959 (5)	0.0251 (15)	0.724 (6)
N54	−0.1486 (6)	0.4743 (3)	−0.0247 (4)	0.0203 (13)*	0.724 (6)
C51	0.4444 (9)	0.4402 (5)	0.7159 (6)	0.035 (2)	0.724 (6)
H51A	0.3809	0.4637	0.7253	0.052*	0.724 (6)
H51B	0.5068	0.4326	0.7682	0.052*	0.724 (6)
H51C	0.4128	0.4013	0.6903	0.052*	0.724 (6)
C52	0.5992 (8)	0.5067 (6)	0.6998 (6)	0.041 (3)	0.724 (6)
H52A	0.5830	0.5503	0.6918	0.061*	0.724 (6)
H52B	0.6571	0.4949	0.6738	0.061*	0.724 (6)
H52C	0.6310	0.4978	0.7584	0.061*	0.724 (6)
C53	0.4320 (7)	0.4775 (4)	0.5818 (5)	0.0188 (16)*	0.724 (6)
C54	0.4811 (8)	0.5084 (4)	0.5292 (6)	0.0262 (17)	0.724 (6)
H54	0.5577	0.5255	0.5514	0.031*	0.724 (6)
C55	0.4221 (8)	0.5141 (4)	0.4483 (6)	0.0231 (18)*	0.724 (6)
H55	0.4552	0.5378	0.4155	0.028*	0.724 (6)
C56	0.3148 (8)	0.4861 (4)	0.4128 (5)	0.0243 (16)	0.724 (6)
C57	0.2605 (8)	0.4549 (4)	0.4613 (6)	0.0255 (19)*	0.724 (6)
H57	0.1841	0.4378	0.4375	0.031*	0.724 (6)
C58	0.3211 (7)	0.4493 (4)	0.5454 (5)	0.0259 (17)	0.724 (6)
H58	0.2872	0.4262	0.5783	0.031*	0.724 (6)
C59	0.0828 (7)	0.4753 (4)	0.2134 (5)	0.0202 (17)*	0.724 (6)
C510	0.1170 (8)	0.5044 (4)	0.1516 (5)	0.0262 (17)	0.724 (6)
H510	0.1907	0.5248	0.1643	0.031*	0.724 (6)
C511	0.0366 (8)	0.5019 (4)	0.0698 (5)	0.0203 (17)*	0.724 (6)
H511	0.0604	0.5180	0.0269	0.024*	0.724 (6)
C512	−0.0766 (7)	0.4765 (4)	0.0504 (5)	0.0196 (14)	0.724 (6)
C513	−0.1071 (8)	0.4492 (4)	0.1152 (5)	0.0224 (18)*	0.724 (6)
H513	−0.1817	0.4301	0.1039	0.027*	0.724 (6)
C514	−0.0298 (8)	0.4505 (4)	0.1936 (5)	0.0259 (17)	0.724 (6)
H514	−0.0538	0.4338	0.2361	0.031*	0.724 (6)
C515	−0.2662 (8)	0.4479 (4)	−0.0488 (6)	0.0318 (19)	0.724 (6)
H55A	−0.2655	0.4125	−0.0144	0.048*	0.724 (6)
H55B	−0.2901	0.4353	−0.1062	0.048*	0.724 (6)
H55C	−0.3222	0.4781	−0.0420	0.048*	0.724 (6)
C516	−0.1176 (8)	0.5007 (5)	−0.0933 (6)	0.032 (2)	0.724 (6)
H56A	−0.1263	0.5449	−0.0932	0.047*	0.724 (6)
H56B	−0.1703	0.4842	−0.1451	0.047*	0.724 (6)
H56C	−0.0357	0.4904	−0.0871	0.047*	0.724 (6)
O61	0.7398 (8)	0.0396 (4)	0.2128 (7)	0.046 (2)	0.674 (7)
N61	0.9297 (8)	0.0927 (4)	0.5843 (5)	0.061 (3)*	0.674 (7)
N62	0.6883 (7)	0.0680 (4)	0.2551 (5)	0.0270 (17)*	0.674 (7)
N63	0.5853 (10)	0.0893 (5)	0.2278 (8)	0.037 (2)*	0.674 (7)
N64	0.2734 (8)	0.0849 (5)	−0.0971 (6)	0.0320 (19)*	0.674 (7)
C61	0.8824 (10)	0.1239 (6)	0.6478 (8)	0.041 (3)	0.674 (7)
H61A	0.8319	0.1583	0.6220	0.061*	0.674 (7)
H61B	0.9490	0.1385	0.6941	0.061*	0.674 (7)
H61C	0.8364	0.0946	0.6674	0.061*	0.674 (7)
C62	1.0398 (11)	0.0546 (7)	0.6284 (10)	0.050 (3)	0.674 (7)
H62A	1.0236	0.0285	0.6691	0.075*	0.674 (7)
H62B	1.1060	0.0816	0.6556	0.075*	0.674 (7)
H62C	1.0601	0.0294	0.5884	0.075*	0.674 (7)
C63	0.8699 (9)	0.0878 (5)	0.5080 (6)	0.030 (2)*	0.674 (7)
C64	0.9224 (10)	0.0607 (5)	0.4530 (8)	0.033 (2)*	0.674 (7)
H64	1.0019	0.0471	0.4737	0.040*	0.674 (7)
C65	0.8603 (9)	0.0541 (5)	0.3712 (7)	0.032 (2)*	0.674 (7)
H65	0.8948	0.0333	0.3367	0.039*	0.674 (7)
C66	0.7487 (11)	0.0775 (6)	0.3393 (8)	0.036 (2)*	0.674 (7)
C67	0.6967 (11)	0.1085 (5)	0.3937 (9)	0.040 (3)	0.674 (7)
H67	0.6186	0.1240	0.3725	0.048*	0.674 (7)
C68	0.7587 (10)	0.1151 (5)	0.4735 (8)	0.031 (2)*	0.674 (7)
H68	0.7269	0.1386	0.5071	0.037*	0.674 (7)
C69	0.5198 (8)	0.0830 (5)	0.1439 (7)	0.028 (2)	0.674 (7)
C610	0.5435 (9)	0.0536 (4)	0.0783 (7)	0.0233 (18)*	0.674 (7)
H610	0.6143	0.0310	0.0885	0.028*	0.674 (7)
C611	0.4656 (9)	0.0575 (4)	0.0008 (7)	0.028 (2)	0.674 (7)
H611	0.4904	0.0421	−0.0420	0.033*	0.674 (7)
C612	0.3493 (8)	0.0829 (4)	−0.0215 (6)	0.0202 (16)*	0.674 (7)
C613	0.3270 (9)	0.1098 (5)	0.0460 (6)	0.0243 (18)*	0.674 (7)
H613	0.2539	0.1301	0.0366	0.029*	0.674 (7)
C614	0.4052 (10)	0.1081 (5)	0.1241 (7)	0.0257 (19)*	0.674 (7)
H614	0.3816	0.1245	0.1669	0.031*	0.674 (7)
C615	0.1567 (8)	0.1132 (5)	−0.1147 (7)	0.030 (2)	0.674 (7)
H65A	0.1219	0.1001	−0.0737	0.045*	0.674 (7)
H65B	0.1053	0.1008	−0.1692	0.045*	0.674 (7)
H65C	0.1653	0.1574	−0.1128	0.045*	0.674 (7)
C616	0.3071 (12)	0.0613 (6)	−0.1642 (9)	0.044 (3)	0.674 (7)
H66A	0.3704	0.0864	−0.1713	0.066*	0.674 (7)
H66B	0.2387	0.0621	−0.2145	0.066*	0.674 (7)
H66C	0.3351	0.0195	−0.1521	0.066*	0.674 (7)
O71	0.7848 (15)	0.0407 (8)	0.2568 (11)	0.0392 (16)*	0.326 (7)
N71	0.9709 (17)	0.0889 (9)	0.6398 (12)	0.0392 (16)*	0.326 (7)
N72	0.7342 (18)	0.0704 (9)	0.3016 (14)	0.0392 (16)*	0.326 (7)
N73	0.6248 (18)	0.0929 (10)	0.2716 (13)	0.0392 (16)*	0.326 (7)
N74	0.3176 (19)	0.0895 (10)	−0.0585 (15)	0.0392 (16)*	0.326 (7)
C71	0.928 (2)	0.1216 (11)	0.6991 (15)	0.0392 (16)*	0.326 (7)
C72	1.085 (2)	0.0566 (11)	0.6823 (15)	0.0392 (16)*	0.326 (7)
C73	0.9297 (8)	0.0927 (4)	0.5843 (5)	0.0392 (16)*	0.326 (7)
C74	0.955 (2)	0.0575 (11)	0.4994 (15)	0.0392 (16)*	0.326 (7)
C75	0.897 (2)	0.0515 (11)	0.4145 (18)	0.0392 (16)*	0.326 (7)
C76	0.784 (2)	0.0811 (11)	0.3878 (15)	0.0392 (16)*	0.326 (7)
C77	0.744 (2)	0.1132 (12)	0.439 (2)	0.0392 (16)*	0.326 (7)
C78	0.803 (2)	0.1176 (11)	0.5202 (15)	0.0392 (16)*	0.326 (7)
C79	0.566 (2)	0.0846 (12)	0.1887 (19)	0.0392 (16)*	0.326 (7)
C710	0.579 (2)	0.0576 (12)	0.1158 (17)	0.0392 (16)*	0.326 (7)
C711	0.517 (2)	0.0525 (12)	0.0420 (19)	0.0392 (16)*	0.326 (7)
C712	0.399 (2)	0.0862 (11)	0.0239 (14)	0.0392 (16)*	0.326 (7)
C713	0.369 (2)	0.1109 (11)	0.0872 (18)	0.0392 (16)*	0.326 (7)
C714	0.451 (2)	0.1110 (11)	0.1669 (15)	0.0392 (16)*	0.326 (7)
C715	0.205 (2)	0.1199 (11)	−0.0706 (15)	0.0392 (16)*	0.326 (7)
C716	0.355 (2)	0.0676 (12)	−0.1252 (16)	0.0392 (16)*	0.326 (7)
O81	0.2568 (15)	0.5270 (8)	0.2269 (11)	0.0332 (15)*	0.276 (6)
N81	0.450 (2)	0.4766 (11)	0.6105 (17)	0.0332 (15)*	0.276 (6)
N82	0.1979 (18)	0.4941 (10)	0.2666 (13)	0.0332 (15)*	0.276 (6)
N83	0.102 (2)	0.4712 (11)	0.2388 (16)	0.0332 (15)*	0.276 (6)
N84	−0.2127 (18)	0.4776 (10)	−0.0865 (12)	0.0332 (15)*	0.276 (6)
C81	0.396 (2)	0.4413 (12)	0.6636 (16)	0.0332 (15)*	0.276 (6)
C82	0.548 (2)	0.5087 (11)	0.6387 (15)	0.0332 (15)*	0.276 (6)
C83	0.378 (2)	0.4779 (12)	0.5188 (15)	0.0332 (15)*	0.276 (6)
C84	0.443 (3)	0.5115 (13)	0.478 (2)	0.0332 (15)*	0.276 (6)
C85	0.368 (2)	0.5129 (11)	0.3862 (15)	0.0332 (15)*	0.276 (6)
C86	0.270 (2)	0.4881 (12)	0.3595 (19)	0.0332 (15)*	0.276 (6)
C87	0.214 (2)	0.4570 (12)	0.4066 (15)	0.0332 (15)*	0.276 (6)
C88	0.275 (2)	0.4508 (13)	0.492 (2)	0.0332 (15)*	0.276 (6)
C89	0.028 (2)	0.4783 (12)	0.1540 (15)	0.0332 (15)*	0.276 (6)
C810	0.065 (3)	0.5061 (13)	0.0922 (19)	0.0332 (15)*	0.276 (6)
C811	−0.025 (2)	0.5085 (11)	0.0060 (15)	0.0332 (15)*	0.276 (6)
C813	−0.167 (2)	0.4549 (11)	0.0545 (15)	0.0332 (15)*	0.276 (6)
C814	−0.075 (3)	0.4494 (13)	0.139 (2)	0.0332 (15)*	0.276 (6)
C815	−0.326 (2)	0.4524 (11)	−0.1049 (15)	0.0332 (15)*	0.276 (6)
C816	−0.184 (2)	0.5034 (11)	−0.1507 (15)	0.0332 (15)*	0.276 (6)

### Atomic displacement parameters (Å^2^)

**Table:**

	*U*^11^	*U*^22^	*U*^33^	*U*^12^	*U*^13^	*U*^23^
O11	0.027 (2)	0.039 (3)	0.035 (2)	−0.010 (2)	0.0199 (19)	0.003 (2)
N11	0.020 (2)	0.033 (3)	0.034 (3)	0.001 (2)	0.011 (2)	−0.002 (2)
N12	0.033 (3)	0.022 (2)	0.046 (3)	0.001 (2)	0.028 (2)	0.003 (2)
N13	0.023 (2)	0.022 (2)	0.031 (2)	0.0015 (19)	0.0146 (19)	0.0025 (19)
C11	0.030 (3)	0.041 (4)	0.035 (3)	0.000 (3)	0.015 (3)	0.006 (3)
C12	0.025 (3)	0.042 (4)	0.040 (3)	−0.006 (3)	0.006 (3)	−0.002 (3)
C13	0.020 (3)	0.027 (3)	0.038 (3)	0.005 (2)	0.014 (2)	0.007 (3)
C14	0.023 (3)	0.026 (3)	0.037 (3)	0.003 (2)	0.015 (2)	0.000 (2)
C15	0.031 (3)	0.029 (3)	0.036 (3)	0.006 (3)	0.016 (3)	0.009 (3)
C16	0.025 (3)	0.016 (3)	0.036 (3)	0.002 (2)	0.016 (2)	−0.002 (2)
C17	0.023 (3)	0.028 (3)	0.031 (3)	0.000 (2)	0.012 (2)	0.003 (2)
C18	0.022 (3)	0.026 (3)	0.035 (3)	0.001 (2)	0.012 (2)	−0.001 (2)
C113	0.026 (3)	0.029 (3)	0.036 (3)	−0.010 (2)	0.020 (3)	−0.008 (3)
C114	0.034 (3)	0.021 (3)	0.035 (3)	0.003 (2)	0.018 (3)	0.003 (2)
C116	0.038 (4)	0.038 (4)	0.031 (3)	0.000 (3)	0.018 (3)	0.002 (3)
O21	0.028 (2)	0.041 (3)	0.049 (3)	0.004 (2)	0.026 (2)	−0.006 (2)
N21	0.029 (3)	0.037 (3)	0.048 (3)	0.008 (2)	0.023 (2)	0.007 (3)
N23	0.034 (3)	0.019 (2)	0.057 (4)	0.005 (2)	0.036 (3)	0.001 (2)
N24	0.031 (3)	0.039 (3)	0.039 (3)	0.005 (2)	0.020 (2)	−0.006 (2)
C21	0.040 (4)	0.037 (4)	0.054 (4)	0.006 (3)	0.030 (3)	0.002 (3)
C22	0.033 (4)	0.047 (5)	0.058 (5)	0.010 (3)	0.024 (3)	0.004 (4)
C23	0.027 (3)	0.021 (3)	0.053 (4)	−0.004 (2)	0.029 (3)	−0.003 (2)
C24	0.033 (3)	0.033 (3)	0.047 (4)	−0.001 (3)	0.028 (3)	0.003 (3)
C26	0.042 (3)	0.017 (3)	0.037 (3)	−0.007 (2)	0.025 (3)	−0.002 (2)
C27	0.032 (3)	0.027 (3)	0.046 (4)	0.003 (3)	0.023 (3)	0.002 (3)
C28	0.033 (3)	0.023 (3)	0.051 (4)	0.003 (2)	0.026 (3)	0.004 (3)
C29	0.034 (3)	0.024 (3)	0.039 (3)	−0.001 (3)	0.021 (3)	0.007 (3)
C210	0.029 (3)	0.024 (3)	0.056 (4)	−0.007 (2)	0.022 (3)	−0.003 (3)
C211	0.027 (3)	0.029 (3)	0.048 (4)	0.004 (3)	0.023 (3)	0.005 (3)
C214	0.037 (3)	0.024 (3)	0.041 (3)	−0.002 (2)	0.022 (3)	−0.001 (3)
C213	0.033 (3)	0.025 (3)	0.044 (3)	0.011 (2)	0.028 (3)	0.009 (3)
C216	0.044 (4)	0.043 (5)	0.047 (4)	0.005 (4)	0.027 (3)	−0.010 (3)
O31	0.040 (3)	0.040 (3)	0.056 (3)	−0.008 (2)	0.032 (3)	0.007 (2)
N31	0.045 (4)	0.048 (4)	0.072 (5)	−0.003 (3)	0.032 (4)	0.014 (4)
N33	0.059 (5)	0.032 (4)	0.106 (7)	−0.018 (3)	0.064 (5)	−0.016 (4)
N34	0.024 (3)	0.042 (3)	0.060 (4)	−0.006 (2)	0.021 (3)	0.006 (3)
C31	0.056 (5)	0.042 (5)	0.077 (6)	0.010 (4)	0.046 (5)	0.015 (4)
C32	0.041 (4)	0.051 (5)	0.078 (6)	−0.003 (4)	0.033 (4)	0.001 (5)
C33	0.037 (4)	0.030 (4)	0.057 (5)	0.004 (3)	0.027 (3)	−0.008 (3)
C34	0.033 (4)	0.036 (4)	0.066 (5)	0.004 (3)	0.028 (4)	0.011 (4)
C35	0.041 (4)	0.031 (4)	0.062 (5)	0.007 (3)	0.032 (4)	0.003 (3)
C36	0.044 (4)	0.040 (4)	0.059 (5)	0.013 (4)	0.025 (4)	−0.003 (4)
C37	0.038 (4)	0.030 (4)	0.066 (5)	0.005 (3)	0.021 (4)	−0.012 (3)
C38	0.035 (4)	0.029 (4)	0.074 (5)	0.004 (3)	0.036 (4)	0.004 (3)
C39	0.047 (4)	0.025 (3)	0.059 (5)	0.009 (3)	0.026 (4)	−0.009 (3)
C310	0.030 (3)	0.025 (3)	0.077 (5)	−0.010 (2)	0.032 (3)	−0.024 (3)
C311	0.032 (3)	0.026 (3)	0.068 (5)	0.002 (2)	0.037 (3)	0.000 (3)
C312	0.042 (4)	0.022 (3)	0.084 (6)	−0.004 (3)	0.041 (4)	−0.009 (3)
C313	0.038 (4)	0.019 (3)	0.075 (6)	0.002 (3)	0.034 (4)	−0.004 (3)
C314	0.053 (5)	0.027 (4)	0.072 (6)	−0.006 (3)	0.044 (4)	−0.007 (3)
C315	0.032 (3)	0.045 (4)	0.078 (6)	−0.015 (3)	0.029 (4)	−0.012 (4)
C316	0.039 (4)	0.059 (6)	0.060 (5)	−0.009 (4)	0.029 (4)	−0.002 (4)
O41	0.039 (3)	0.043 (3)	0.069 (4)	0.006 (3)	0.039 (3)	−0.004 (3)
N41	0.035 (3)	0.048 (4)	0.051 (4)	0.005 (3)	0.022 (3)	−0.005 (3)
N42	0.054 (4)	0.027 (3)	0.081 (5)	0.001 (3)	0.054 (4)	0.000 (3)
N43	0.056 (4)	0.027 (3)	0.081 (5)	0.009 (3)	0.047 (4)	0.003 (3)
N44	0.035 (3)	0.035 (3)	0.063 (4)	0.000 (2)	0.029 (3)	−0.015 (3)
C41	0.040 (4)	0.037 (4)	0.049 (4)	0.006 (3)	0.024 (3)	0.003 (3)
C42	0.031 (3)	0.046 (5)	0.061 (5)	0.009 (3)	0.025 (3)	0.001 (4)
C43	0.032 (3)	0.028 (3)	0.052 (4)	−0.005 (2)	0.026 (3)	0.002 (3)
C44	0.040 (4)	0.041 (4)	0.062 (5)	0.000 (3)	0.036 (4)	−0.005 (4)
C45	0.051 (4)	0.038 (4)	0.070 (6)	−0.003 (3)	0.044 (4)	0.009 (4)
C46	0.045 (4)	0.032 (4)	0.052 (4)	−0.008 (3)	0.029 (3)	0.002 (3)
C47	0.040 (4)	0.032 (4)	0.064 (5)	−0.001 (3)	0.028 (4)	0.011 (3)
C48	0.031 (3)	0.028 (3)	0.058 (4)	−0.001 (3)	0.028 (3)	−0.003 (3)
C49	0.047 (4)	0.023 (3)	0.066 (5)	−0.009 (3)	0.026 (4)	0.005 (3)
C410	0.025 (3)	0.027 (4)	0.075 (5)	0.005 (3)	0.026 (3)	0.014 (3)
C411	0.038 (4)	0.022 (3)	0.081 (6)	−0.007 (3)	0.044 (4)	−0.007 (3)
C412	0.033 (3)	0.022 (3)	0.061 (4)	0.003 (3)	0.034 (3)	0.003 (3)
C413	0.040 (4)	0.019 (3)	0.071 (5)	−0.006 (3)	0.039 (4)	−0.003 (3)
C414	0.048 (4)	0.031 (4)	0.050 (4)	0.003 (3)	0.031 (3)	0.008 (3)
O51	0.035 (3)	0.035 (4)	0.040 (4)	−0.009 (3)	0.025 (3)	0.000 (3)
N51	0.023 (3)	0.030 (4)	0.030 (4)	−0.001 (3)	0.008 (3)	0.003 (3)
N53	0.019 (3)	0.027 (4)	0.037 (4)	0.005 (3)	0.019 (3)	0.000 (3)
C51	0.033 (5)	0.037 (5)	0.040 (5)	0.003 (4)	0.021 (4)	0.009 (4)
C52	0.023 (4)	0.057 (7)	0.039 (5)	0.004 (4)	0.005 (4)	0.004 (5)
C54	0.025 (4)	0.018 (4)	0.036 (5)	0.002 (3)	0.011 (3)	0.000 (3)
C56	0.027 (4)	0.020 (4)	0.029 (4)	0.007 (3)	0.012 (3)	0.001 (3)
C58	0.022 (4)	0.020 (4)	0.035 (4)	0.000 (3)	0.009 (3)	−0.001 (3)
C510	0.032 (4)	0.019 (4)	0.032 (4)	0.003 (3)	0.017 (3)	−0.005 (3)
C512	0.026 (4)	0.013 (3)	0.025 (3)	0.005 (3)	0.016 (3)	−0.001 (3)
C514	0.031 (4)	0.020 (4)	0.032 (4)	0.004 (3)	0.018 (3)	0.001 (3)
C515	0.026 (4)	0.023 (4)	0.055 (5)	−0.001 (3)	0.024 (4)	−0.001 (4)
C516	0.034 (4)	0.033 (4)	0.038 (4)	0.006 (3)	0.026 (4)	0.012 (3)
O61	0.038 (4)	0.039 (5)	0.074 (6)	0.013 (4)	0.038 (4)	−0.001 (4)
C61	0.031 (5)	0.033 (6)	0.065 (8)	−0.008 (4)	0.025 (5)	−0.011 (5)
C62	0.031 (6)	0.043 (7)	0.080 (10)	0.000 (5)	0.024 (6)	0.002 (7)
C67	0.034 (5)	0.025 (5)	0.059 (7)	−0.014 (4)	0.012 (5)	0.001 (5)
C69	0.020 (4)	0.021 (4)	0.044 (6)	−0.002 (3)	0.013 (4)	0.012 (4)
C611	0.026 (4)	0.015 (4)	0.054 (6)	0.011 (3)	0.029 (5)	0.016 (4)
C615	0.022 (4)	0.032 (5)	0.043 (5)	0.006 (3)	0.021 (4)	0.004 (4)
C616	0.044 (6)	0.047 (7)	0.055 (7)	0.000 (6)	0.037 (6)	−0.005 (6)

### Geometric parameters (Å, °)

**Table:**

O11—N12	1.297 (7)	C44—H44	0.9500
N11—C13	1.381 (8)	C45—C46	1.418 (12)
N11—C12	1.414 (9)	C45—H45	0.9500
N11—C11	1.456 (9)	C46—C47	1.371 (11)
N12—N13	1.266 (8)	C47—C48	1.359 (12)
N12—C16	1.475 (9)	C47—H47	0.9500
N13—C19	1.409 (8)	C48—H48	0.9500
N14—C112	1.373 (8)	C49—C410	1.368 (12)
N14—C115	1.422 (9)	C49—C414	1.402 (12)
N14—C116	1.453 (9)	C410—C411	1.362 (13)
C11—H11A	0.9800	C410—H410	0.9500
C11—H11B	0.9800	C411—C412	1.420 (10)
C11—H11C	0.9800	C411—H411	0.9500
C12—H12A	0.9800	C412—C413	1.392 (10)
C12—H12B	0.9800	C413—C414	1.398 (12)
C12—H12C	0.9800	C413—H413	0.9500
C13—C18	1.402 (9)	C414—H414	0.9500
C13—C14	1.428 (9)	C415—H45A	0.9800
C14—C15	1.376 (10)	C415—H45B	0.9800
C14—H14	0.9500	C415—H45C	0.9800
C15—C16	1.354 (9)	C416—H46A	0.9800
C15—H15	0.9500	C416—H46B	0.9800
C16—C17	1.403 (9)	C416—H46C	0.9800
C17—C18	1.390 (9)	O51—N52	1.321 (10)
C17—H17	0.9500	N51—C53	1.361 (10)
C18—H18	0.9500	N51—C51	1.422 (12)
C19—C114	1.411 (9)	N51—C52	1.439 (13)
C19—C110	1.424 (9)	N52—N53	1.287 (10)
C110—C111	1.399 (8)	N52—C56	1.429 (11)
C110—H110	0.9500	N53—C59	1.392 (10)
C111—C112	1.429 (9)	N54—C512	1.303 (10)
C111—H111	0.9500	N54—C515	1.454 (11)
C112—C113	1.411 (9)	N54—C516	1.478 (10)
C113—C114	1.369 (10)	C51—H51A	0.9800
C113—H113	0.9500	C51—H51B	0.9800
C114—H114	0.9500	C51—H51C	0.9800
C115—H15A	0.9800	C52—H52A	0.9800
C115—H15B	0.9800	C52—H52B	0.9800
C115—H15C	0.9800	C52—H52C	0.9800
C116—H16A	0.9800	C53—C54	1.414 (12)
C116—H16B	0.9800	C53—C58	1.415 (11)
C116—H16C	0.9800	C54—C55	1.350 (12)
O21—N22	1.303 (8)	C54—H54	0.9500
N21—C23	1.351 (10)	C55—C56	1.375 (12)
N21—C22	1.414 (10)	C55—H55	0.9500
N21—C21	1.447 (10)	C56—C57	1.403 (13)
N22—N23	1.294 (9)	C57—C58	1.402 (12)
N22—C26	1.450 (9)	C57—H57	0.9500
N23—C29	1.436 (10)	C58—H58	0.9500
N24—C212	1.381 (9)	C59—C514	1.392 (12)
N24—C215	1.423 (10)	C59—C510	1.421 (12)
N24—C216	1.456 (9)	C510—C511	1.427 (12)
C21—H21A	0.9800	C510—H510	0.9500
C21—H21B	0.9800	C511—C512	1.403 (12)
C21—H21C	0.9800	C511—H511	0.9500
C22—H22A	0.9800	C512—C513	1.425 (11)
C22—H22B	0.9800	C513—C514	1.367 (12)
C22—H22C	0.9800	C513—H513	0.9500
C23—C24	1.419 (9)	C514—H514	0.9500
C23—C28	1.424 (10)	C515—H55A	0.9800
C24—C25	1.371 (10)	C515—H55B	0.9800
C24—H24	0.9500	C515—H55C	0.9800
C25—C26	1.364 (10)	C516—H56A	0.9800
C25—H25	0.9500	C516—H56B	0.9800
C26—C27	1.396 (9)	C516—H56C	0.9800
C27—C28	1.385 (10)	O61—N62	1.270 (12)
C27—H27	0.9500	N61—C63	1.282 (13)
C28—H28	0.9500	N61—C62	1.538 (16)
C29—C210	1.404 (10)	N61—C61	1.558 (15)
C29—C214	1.406 (10)	N62—N63	1.259 (14)
C210—C211	1.402 (11)	N62—C66	1.413 (14)
C210—H210	0.9500	N63—C69	1.413 (14)
C211—C212	1.423 (9)	N64—C612	1.325 (12)
C211—H211	0.9500	N64—C616	1.449 (15)
C212—C213	1.415 (9)	N64—C615	1.469 (13)
C214—C213	1.381 (10)	C61—H61A	0.9800
C214—H214	0.9500	C61—H61B	0.9800
C213—H213	0.9500	C61—H61C	0.9800
C215—H25A	0.9800	C62—H62A	0.9800
C215—H25B	0.9800	C62—H62B	0.9800
C215—H25C	0.9800	C62—H62C	0.9800
C216—H26A	0.9800	C63—C68	1.404 (15)
C216—H26B	0.9800	C63—C64	1.436 (16)
C216—H26C	0.9800	C64—C65	1.374 (14)
O31—N32	1.252 (8)	C64—H64	0.9500
N31—C33	1.384 (12)	C65—C66	1.369 (16)
N31—C31	1.444 (11)	C65—H65	0.9500
N31—C32	1.482 (12)	C66—C67	1.462 (18)
N32—N33	1.241 (10)	C67—C68	1.344 (16)
N32—C36	1.476 (11)	C67—H67	0.9500
N33—C39	1.501 (13)	C68—H68	0.9500
N34—C312	1.390 (12)	C69—C614	1.416 (14)
N34—C316	1.448 (11)	C69—C610	1.417 (15)
N34—C315	1.472 (9)	C610—C611	1.360 (15)
C31—H31A	0.9800	C610—H610	0.9500
C31—H31B	0.9800	C611—C612	1.432 (12)
C31—H31C	0.9800	C611—H611	0.9500
C32—H32A	0.9800	C612—C613	1.415 (13)
C32—H32B	0.9800	C613—C614	1.367 (13)
C32—H32C	0.9800	C613—H613	0.9500
C33—C34	1.383 (11)	C614—H614	0.9500
C33—C38	1.407 (11)	C615—H65A	0.9800
C34—C35	1.421 (13)	C615—H65B	0.9800
C34—H34	0.9500	C615—H65C	0.9800
C35—C36	1.397 (12)	C616—H66A	0.9800
C35—H35	0.9500	C616—H66B	0.9800
C36—C37	1.390 (13)	C616—H66C	0.9800
C37—C38	1.381 (12)	O71—N72	1.31 (3)
C37—H37	0.9500	N71—C71	1.48 (3)
C38—H38	0.9500	N71—C72	1.51 (3)
C39—C314	1.394 (12)	N72—N73	1.34 (3)
C39—C310	1.398 (12)	N72—C76	1.43 (3)
C310—C311	1.369 (12)	N73—C79	1.38 (3)
C310—H310	0.9500	N74—C712	1.44 (3)
C311—C312	1.385 (10)	N74—C716	1.46 (3)
C311—H311	0.9500	N74—C715	1.46 (3)
C312—C313	1.393 (12)	C74—C75	1.41 (3)
C313—C314	1.389 (13)	C75—C76	1.44 (3)
C313—H313	0.9500	C76—C77	1.34 (4)
C314—H314	0.9500	C77—C78	1.36 (4)
C315—H35A	0.9800	C79—C714	1.43 (3)
C315—H35B	0.9800	C79—C710	1.45 (4)
C315—H35C	0.9800	C710—C711	1.25 (3)
C316—H36A	0.9800	C711—C712	1.53 (3)
C316—H36B	0.9800	C712—C713	1.38 (3)
C316—H36C	0.9800	C713—C714	1.40 (3)
O41—N42	1.248 (8)	O81—N82	1.35 (3)
N41—C43	1.384 (10)	N81—C82	1.32 (3)
N41—C41	1.446 (10)	N81—C81	1.51 (3)
N41—C42	1.469 (10)	N81—C83	1.54 (4)
N42—N43	1.242 (10)	N82—N83	1.20 (3)
N42—C46	1.502 (12)	N82—C86	1.56 (4)
N43—C49	1.474 (12)	N83—C89	1.45 (3)
N44—C412	1.375 (10)	N84—C816	1.39 (3)
N44—C416	1.442 (11)	N84—C815	1.40 (3)
N44—C415	1.457 (10)	C83—C88	1.31 (4)
C41—H41A	0.9800	C83—C84	1.42 (4)
C41—H41B	0.9800	C84—C85	1.55 (4)
C41—H41C	0.9800	C85—C86	1.24 (4)
C42—H42A	0.9800	C86—C87	1.40 (4)
C42—H42B	0.9800	C87—C88	1.42 (4)
C42—H42C	0.9800	C89—C814	1.33 (4)
C43—C44	1.392 (10)	C89—C810	1.43 (4)
C43—C48	1.416 (10)	C810—C811	1.53 (4)
C44—C45	1.406 (13)	C813—C814	1.52 (4)
			
C13—N11—C12	121.4 (6)	C48—C47—H47	119.1
C13—N11—C11	120.0 (6)	C46—C47—H47	119.1
C12—N11—C11	118.5 (6)	C47—C48—C43	120.2 (7)
N13—N12—O11	128.2 (6)	C47—C48—H48	119.9
N13—N12—C16	116.1 (5)	C43—C48—H48	119.9
O11—N12—C16	115.7 (5)	C410—C49—C414	118.0 (8)
N12—N13—C19	119.6 (5)	C410—C49—N43	133.9 (8)
C112—N14—C115	121.8 (6)	C414—C49—N43	108.1 (7)
C112—N14—C116	121.0 (6)	C411—C410—C49	123.2 (7)
C115—N14—C116	117.0 (6)	C411—C410—H410	118.4
N11—C13—C18	122.4 (6)	C49—C410—H410	118.4
N11—C13—C14	119.5 (6)	C410—C411—C412	120.0 (7)
C18—C13—C14	118.0 (6)	C410—C411—H411	120.0
C15—C14—C13	119.3 (6)	C412—C411—H411	120.0
C15—C14—H14	120.4	N44—C412—C413	121.7 (7)
C13—C14—H14	120.4	N44—C412—C411	121.1 (7)
C16—C15—C14	122.8 (7)	C413—C412—C411	117.0 (8)
C16—C15—H15	118.6	C412—C413—C414	121.5 (7)
C14—C15—H15	118.6	C412—C413—H413	119.2
C15—C16—C17	119.1 (6)	C414—C413—H413	119.2
C15—C16—N12	119.7 (6)	C413—C414—C49	119.8 (8)
C17—C16—N12	121.2 (6)	C413—C414—H414	120.1
C18—C17—C16	120.2 (6)	C49—C414—H414	120.1
C18—C17—H17	119.9	C53—N51—C51	121.2 (8)
C16—C17—H17	119.9	C53—N51—C52	120.2 (8)
C17—C18—C13	120.6 (6)	C51—N51—C52	118.3 (8)
C17—C18—H18	119.7	N53—N52—O51	123.4 (8)
C13—C18—H18	119.7	N53—N52—C56	119.2 (8)
N13—C19—C114	112.4 (6)	O51—N52—C56	117.4 (7)
N13—C19—C110	129.9 (6)	N52—N53—C59	123.2 (7)
C114—C19—C110	117.6 (6)	C512—N54—C515	124.0 (7)
C111—C110—C19	121.0 (6)	C512—N54—C516	122.0 (7)
C111—C110—H110	119.5	C515—N54—C516	114.0 (7)
C19—C110—H110	119.5	N51—C53—C54	121.2 (8)
C110—C111—C112	119.9 (6)	N51—C53—C58	121.6 (8)
C110—C111—H111	120.0	C54—C53—C58	117.1 (8)
C112—C111—H111	120.0	C55—C54—C53	121.9 (8)
N14—C112—C113	121.6 (6)	C55—C54—H54	119.0
N14—C112—C111	120.3 (6)	C53—C54—H54	119.0
C113—C112—C111	118.0 (5)	C54—C55—C56	120.8 (9)
C114—C113—C112	121.3 (6)	C54—C55—H55	119.6
C114—C113—H113	119.4	C56—C55—H55	119.6
C112—C113—H113	119.4	C55—C56—C57	120.3 (8)
C113—C114—C19	121.7 (6)	C55—C56—N52	119.9 (8)
C113—C114—H114	119.1	C57—C56—N52	119.6 (8)
C19—C114—H114	119.1	C58—C57—C56	118.9 (8)
C23—N21—C22	119.9 (6)	C58—C57—H57	120.6
C23—N21—C21	120.8 (6)	C56—C57—H57	120.6
C22—N21—C21	119.2 (7)	C57—C58—C53	120.7 (8)
N23—N22—O21	124.9 (6)	C57—C58—H58	119.6
N23—N22—C26	115.8 (6)	C53—C58—H58	119.6
O21—N22—C26	119.2 (6)	N53—C59—C514	113.7 (8)
N22—N23—C29	120.0 (6)	N53—C59—C510	126.8 (8)
C212—N24—C215	122.1 (6)	C514—C59—C510	119.5 (8)
C212—N24—C216	121.1 (6)	C59—C510—C511	117.5 (8)
C215—N24—C216	116.5 (6)	C59—C510—H510	121.3
N21—C23—C24	120.2 (6)	C511—C510—H510	121.3
N21—C23—C28	121.9 (6)	C512—C511—C510	122.3 (8)
C24—C23—C28	117.6 (7)	C512—C511—H511	118.8
C25—C24—C23	120.3 (7)	C510—C511—H511	118.8
C25—C24—H24	119.9	N54—C512—C511	121.4 (7)
C23—C24—H24	119.9	N54—C512—C513	121.0 (8)
C26—C25—C24	121.2 (7)	C511—C512—C513	117.5 (8)
C26—C25—H25	119.4	C514—C513—C512	120.5 (8)
C24—C25—H25	119.4	C514—C513—H513	119.7
C25—C26—C27	120.8 (7)	C512—C513—H513	119.7
C25—C26—N22	118.9 (6)	C513—C514—C59	122.4 (8)
C27—C26—N22	120.4 (7)	C513—C514—H514	118.8
C28—C27—C26	119.3 (7)	C59—C514—H514	118.8
C28—C27—H27	120.3	C63—N61—C62	124.1 (10)
C26—C27—H27	120.3	C63—N61—C61	123.8 (10)
C27—C28—C23	120.6 (7)	C62—N61—C61	110.2 (10)
C27—C28—H28	119.7	N63—N62—O61	124.9 (10)
C23—C28—H28	119.7	N63—N62—C66	115.8 (10)
C210—C29—C214	117.9 (7)	O61—N62—C66	119.3 (10)
C210—C29—N23	129.7 (6)	N62—N63—C69	119.4 (10)
C214—C29—N23	112.1 (6)	C612—N64—C616	119.9 (10)
C211—C210—C29	121.5 (7)	C612—N64—C615	120.9 (9)
C211—C210—H210	119.3	C616—N64—C615	119.2 (10)
C29—C210—H210	119.3	N61—C63—C68	122.0 (10)
C210—C211—C212	119.8 (7)	N61—C63—C64	120.1 (10)
C210—C211—H211	120.1	C68—C63—C64	117.4 (10)
C212—C211—H211	120.1	C65—C64—C63	121.5 (10)
N24—C212—C213	121.2 (6)	C65—C64—H64	119.2
N24—C212—C211	120.9 (6)	C63—C64—H64	119.2
C213—C212—C211	117.7 (6)	C66—C65—C64	119.9 (11)
C213—C214—C29	121.2 (7)	C66—C65—H65	120.0
C213—C214—H214	119.4	C64—C65—H65	120.0
C29—C214—H214	119.4	C65—C66—N62	117.2 (11)
C214—C213—C212	121.3 (6)	C65—C66—C67	119.1 (11)
C214—C213—H213	119.4	N62—C66—C67	123.6 (11)
C212—C213—H213	119.4	C68—C67—C66	120.4 (12)
C33—N31—C31	122.3 (8)	C68—C67—H67	119.8
C33—N31—C32	120.5 (7)	C66—C67—H67	119.8
C31—N31—C32	117.0 (9)	C67—C68—C63	121.0 (12)
N33—N32—O31	129.3 (8)	C67—C68—H68	119.5
N33—N32—C36	111.3 (8)	C63—C68—H68	119.5
O31—N32—C36	119.3 (7)	N63—C69—C614	112.0 (10)
N32—N33—C39	115.3 (7)	N63—C69—C610	132.9 (10)
C312—N34—C316	120.3 (6)	C614—C69—C610	115.0 (10)
C312—N34—C315	120.8 (7)	C611—C610—C69	120.5 (9)
C316—N34—C315	118.6 (7)	C611—C610—H610	119.7
C34—C33—N31	121.7 (8)	C69—C610—H610	119.7
C34—C33—C38	118.2 (8)	C610—C611—C612	125.4 (9)
N31—C33—C38	120.1 (8)	C610—C611—H611	117.3
C33—C34—C35	122.9 (8)	C612—C611—H611	117.3
C33—C34—H34	118.6	N64—C612—C613	123.2 (9)
C35—C34—H34	118.6	N64—C612—C611	124.6 (9)
C36—C35—C34	116.2 (8)	C613—C612—C611	112.1 (9)
C36—C35—H35	121.9	C614—C613—C612	123.4 (9)
C34—C35—H35	121.9	C614—C613—H613	118.3
C37—C36—C35	122.1 (9)	C612—C613—H613	118.3
C37—C36—N32	124.5 (8)	C613—C614—C69	122.8 (10)
C35—C36—N32	113.4 (8)	C613—C614—H614	118.6
C38—C37—C36	119.8 (8)	C69—C614—H614	118.6
C38—C37—H37	120.1	C71—N71—C72	111.3 (18)
C36—C37—H37	120.1	O71—N72—N73	123 (2)
C37—C38—C33	120.7 (8)	O71—N72—C76	126 (2)
C37—C38—H38	119.7	N73—N72—C76	111 (2)
C33—C38—H38	119.7	N72—N73—C79	117 (2)
C314—C39—C310	116.9 (8)	C712—N74—C716	119 (2)
C314—C39—N33	109.7 (8)	C712—N74—C715	117 (2)
C310—C39—N33	133.4 (8)	C716—N74—C715	124 (2)
C311—C310—C39	120.5 (7)	C74—C75—C76	112 (2)
C311—C310—H310	119.8	C77—C76—N72	131 (2)
C39—C310—H310	119.8	C77—C76—C75	122 (2)
C310—C311—C312	122.6 (8)	N72—C76—C75	106 (2)
C310—C311—H311	118.7	C76—C77—C78	123 (3)
C312—C311—H311	118.7	N73—C79—C714	109 (2)
C311—C312—N34	122.4 (8)	N73—C79—C710	143 (2)
C311—C312—C313	117.7 (9)	C714—C79—C710	108 (3)
N34—C312—C313	119.8 (7)	C711—C710—C79	136 (3)
C314—C313—C312	119.7 (8)	C710—C711—C712	111 (2)
C314—C313—H313	120.1	C713—C712—N74	120 (2)
C312—C313—H313	120.1	C713—C712—C711	120 (2)
C313—C314—C39	122.3 (8)	N74—C712—C711	120 (2)
C313—C314—H314	118.8	C712—C713—C714	120 (2)
C39—C314—H314	118.8	C713—C714—C79	124 (2)
C43—N41—C41	121.6 (7)	C82—N81—C81	124 (2)
C43—N41—C42	121.2 (7)	C82—N81—C83	120 (2)
C41—N41—C42	117.2 (7)	C81—N81—C83	116 (2)
N43—N42—O41	130.8 (9)	N83—N82—O81	128 (2)
N43—N42—C46	110.0 (7)	N83—N82—C86	121 (2)
O41—N42—C46	119.2 (7)	O81—N82—C86	111.4 (18)
N42—N43—C49	114.4 (7)	N82—N83—C89	123 (2)
C412—N44—C416	120.2 (6)	C816—N84—C815	117 (2)
C412—N44—C415	120.3 (6)	C88—C83—C84	132 (3)
C416—N44—C415	119.3 (7)	C88—C83—N81	120 (2)
N41—C43—C44	120.6 (7)	C84—C83—N81	108 (2)
N41—C43—C48	121.0 (7)	C83—C84—C85	107 (2)
C44—C43—C48	118.1 (8)	C86—C85—C84	122 (3)
C43—C44—C45	122.0 (8)	C85—C86—C87	125 (3)
C43—C44—H44	119.0	C85—C86—N82	119 (2)
C45—C44—H44	119.0	C87—C86—N82	116 (2)
C44—C45—C46	117.1 (7)	C86—C87—C88	119 (2)
C44—C45—H45	121.4	C83—C88—C87	115 (3)
C46—C45—H45	121.4	C814—C89—C810	124 (3)
C47—C46—C45	120.4 (8)	C814—C89—N83	111 (2)
C47—C46—N42	125.9 (8)	C810—C89—N83	124 (2)
C45—C46—N42	113.6 (7)	C89—C810—C811	117 (2)
C48—C47—C46	121.7 (8)	C89—C814—C813	119 (2)
			
O11—N12—N13—C19	−1.1 (10)	C415—N44—C412—C413	2.4 (11)
C16—N12—N13—C19	177.3 (5)	C416—N44—C412—C411	−8.4 (11)
C12—N11—C13—C18	−174.1 (7)	C415—N44—C412—C411	177.0 (7)
C11—N11—C13—C18	4.6 (10)	C410—C411—C412—N44	177.5 (7)
C12—N11—C13—C14	3.5 (10)	C410—C411—C412—C413	−7.6 (10)
C11—N11—C13—C14	−177.8 (6)	N44—C412—C413—C414	−177.8 (7)
N11—C13—C14—C15	−178.4 (6)	C411—C412—C413—C414	7.3 (10)
C18—C13—C14—C15	−0.8 (10)	C412—C413—C414—C49	−5.3 (11)
C13—C14—C15—C16	1.3 (11)	C410—C49—C414—C413	3.2 (11)
C14—C15—C16—C17	−0.9 (10)	N43—C49—C414—C413	179.8 (7)
C14—C15—C16—N12	177.2 (6)	O51—N52—N53—C59	1.0 (13)
N13—N12—C16—C15	−177.2 (6)	C56—N52—N53—C59	179.6 (8)
O11—N12—C16—C15	1.5 (9)	C51—N51—C53—C54	−177.5 (9)
N13—N12—C16—C17	0.9 (9)	C52—N51—C53—C54	8.0 (13)
O11—N12—C16—C17	179.5 (6)	C51—N51—C53—C58	−0.2 (14)
C15—C16—C17—C18	−0.2 (10)	C52—N51—C53—C58	−174.6 (9)
N12—C16—C17—C18	−178.2 (6)	N51—C53—C54—C55	−178.6 (9)
C16—C17—C18—C13	0.7 (10)	C58—C53—C54—C55	4.0 (13)
N11—C13—C18—C17	177.4 (6)	C53—C54—C55—C56	−4.8 (14)
C14—C13—C18—C17	−0.2 (10)	C54—C55—C56—C57	4.9 (14)
N12—N13—C19—C114	−179.4 (6)	C54—C55—C56—N52	179.5 (8)
N12—N13—C19—C110	5.2 (11)	N53—N52—C56—C55	−175.2 (8)
N13—C19—C110—C111	178.7 (7)	O51—N52—C56—C55	3.5 (12)
C114—C19—C110—C111	3.6 (10)	N53—N52—C56—C57	−0.6 (12)
C19—C110—C111—C112	−5.4 (10)	O51—N52—C56—C57	178.1 (8)
C115—N14—C112—C113	1.1 (10)	C55—C56—C57—C58	−4.2 (13)
C116—N14—C112—C113	178.0 (7)	N52—C56—C57—C58	−178.9 (8)
C115—N14—C112—C111	−175.7 (6)	C56—C57—C58—C53	3.6 (14)
C116—N14—C112—C111	1.3 (10)	N51—C53—C58—C57	179.2 (9)
C110—C111—C112—N14	−175.9 (6)	C54—C53—C58—C57	−3.4 (13)
C110—C111—C112—C113	7.2 (10)	N52—N53—C59—C514	−176.3 (8)
N14—C112—C113—C114	175.6 (7)	N52—N53—C59—C510	1.1 (14)
C111—C112—C113—C114	−7.6 (10)	N53—C59—C510—C511	177.3 (9)
C112—C113—C114—C19	6.1 (11)	C514—C59—C510—C511	−5.5 (13)
N13—C19—C114—C113	−179.9 (6)	C59—C510—C511—C512	5.4 (13)
C110—C19—C114—C113	−3.9 (10)	C515—N54—C512—C511	179.2 (8)
O21—N22—N23—C29	−0.5 (10)	C516—N54—C512—C511	−2.6 (12)
C26—N22—N23—C29	176.7 (6)	C515—N54—C512—C513	3.6 (13)
C22—N21—C23—C24	4.5 (10)	C516—N54—C512—C513	−178.2 (8)
C21—N21—C23—C24	−178.9 (7)	C510—C511—C512—N54	−179.6 (8)
C22—N21—C23—C28	−170.0 (7)	C510—C511—C512—C513	−3.9 (12)
C21—N21—C23—C28	6.6 (11)	N54—C512—C513—C514	178.4 (8)
N21—C23—C24—C25	−179.0 (7)	C511—C512—C513—C514	2.7 (13)
C28—C23—C24—C25	−4.3 (10)	C512—C513—C514—C59	−3.1 (14)
C23—C24—C25—C26	3.5 (11)	N53—C59—C514—C513	−177.9 (8)
C24—C25—C26—C27	−2.1 (11)	C510—C59—C514—C513	4.6 (14)
C24—C25—C26—N22	177.7 (7)	O61—N62—N63—C69	−1.0 (17)
N23—N22—C26—C25	−179.2 (6)	C66—N62—N63—C69	178.5 (10)
O21—N22—C26—C25	−1.8 (10)	C62—N61—C63—C68	168.2 (11)
N23—N22—C26—C27	0.6 (9)	C61—N61—C63—C68	5.5 (17)
O21—N22—C26—C27	178.0 (6)	C62—N61—C63—C64	−20.2 (17)
C25—C26—C27—C28	1.6 (10)	C61—N61—C63—C64	177.1 (10)
N22—C26—C27—C28	−178.2 (6)	N61—C63—C64—C65	179.1 (11)
C26—C27—C28—C23	−2.6 (11)	C68—C63—C64—C65	−8.9 (17)
N21—C23—C28—C27	178.5 (7)	C63—C64—C65—C66	4.8 (17)
C24—C23—C28—C27	3.9 (10)	C64—C65—C66—N62	−178.5 (10)
N22—N23—C29—C210	6.6 (11)	C64—C65—C66—C67	−1.0 (17)
N22—N23—C29—C214	−179.2 (6)	N63—N62—C66—C65	179.1 (11)
C214—C29—C210—C211	5.9 (10)	O61—N62—C66—C65	−1.3 (16)
N23—C29—C210—C211	179.8 (7)	N63—N62—C66—C67	1.7 (16)
C29—C210—C211—C212	−7.1 (11)	O61—N62—C66—C67	−178.7 (10)
C215—N24—C212—C213	−3.8 (11)	C65—C66—C67—C68	1.6 (17)
C216—N24—C212—C213	−177.9 (7)	N62—C66—C67—C68	178.9 (11)
C215—N24—C212—C211	−178.0 (7)	C66—C67—C68—C63	−6.0 (17)
C216—N24—C212—C211	7.9 (11)	N61—C63—C68—C67	−178.8 (11)
C210—C211—C212—N24	−178.3 (7)	C64—C63—C68—C67	9.4 (17)
C210—C211—C212—C213	7.3 (10)	N62—N63—C69—C614	178.5 (10)
C210—C29—C214—C213	−5.2 (10)	N62—N63—C69—C610	3.2 (18)
N23—C29—C214—C213	179.8 (6)	N63—C69—C610—C611	−176.3 (11)
C29—C214—C213—C212	5.9 (11)	C614—C69—C610—C611	8.5 (14)
N24—C212—C213—C214	178.8 (7)	C69—C610—C611—C612	−8.9 (15)
C211—C212—C213—C214	−6.8 (11)	C616—N64—C612—C613	174.2 (10)
O31—N32—N33—C39	−2.0 (13)	C615—N64—C612—C613	−3.2 (15)
C36—N32—N33—C39	−179.2 (6)	C616—N64—C612—C611	−1.2 (16)
C31—N31—C33—C34	177.4 (8)	C615—N64—C612—C611	−178.5 (9)
C32—N31—C33—C34	−6.9 (13)	C610—C611—C612—N64	−178.1 (10)
C31—N31—C33—C38	−1.7 (13)	C610—C611—C612—C613	6.0 (14)
C32—N31—C33—C38	174.0 (8)	N64—C612—C613—C614	−179.7 (10)
N31—C33—C34—C35	178.2 (8)	C611—C612—C613—C614	−3.8 (14)
C38—C33—C34—C35	−2.6 (12)	C612—C613—C614—C69	4.7 (16)
C33—C34—C35—C36	−0.6 (12)	N63—C69—C614—C613	177.1 (10)
C34—C35—C36—C37	3.1 (12)	C610—C69—C614—C613	−6.7 (14)
C34—C35—C36—N32	−177.2 (7)	O71—N72—N73—C79	−1(3)
N33—N32—C36—C37	−4.0 (12)	C76—N72—N73—C79	−179 (2)
O31—N32—C36—C37	178.5 (8)	O71—N72—C76—C77	176 (2)
N33—N32—C36—C35	176.4 (7)	N73—N72—C76—C77	−6(4)
O31—N32—C36—C35	−1.1 (11)	O71—N72—C76—C75	−2(3)
C35—C36—C37—C38	−2.3 (12)	N73—N72—C76—C75	175.9 (19)
N32—C36—C37—C38	178.1 (7)	C74—C75—C76—C77	3(3)
C36—C37—C38—C33	−1.2 (12)	C74—C75—C76—N72	−179 (2)
C34—C33—C38—C37	3.6 (12)	N72—C76—C77—C78	179 (2)
N31—C33—C38—C37	−177.3 (8)	C75—C76—C77—C78	−4(4)
N32—N33—C39—C314	179.7 (7)	N72—N73—C79—C714	180 (2)
N32—N33—C39—C310	1.9 (13)	N72—N73—C79—C710	1(5)
C314—C39—C310—C311	5.4 (11)	N73—C79—C710—C711	179 (3)
N33—C39—C310—C311	−177.0 (8)	C714—C79—C710—C711	1(4)
C39—C310—C311—C312	−3.4 (11)	C79—C710—C711—C712	4(4)
C310—C311—C312—N34	176.6 (7)	C716—N74—C712—C713	175 (2)
C310—C311—C312—C313	0.6 (11)	C715—N74—C712—C713	0(3)
C316—N34—C312—C311	3.1 (12)	C716—N74—C712—C711	−8(3)
C315—N34—C312—C311	177.6 (7)	C715—N74—C712—C711	177 (2)
C316—N34—C312—C313	179.1 (8)	C710—C711—C712—C713	−7(3)
C315—N34—C312—C313	−6.4 (11)	C710—C711—C712—N74	176 (2)
C311—C312—C313—C314	0.0 (11)	N74—C712—C713—C714	−176 (2)
N34—C312—C313—C314	−176.1 (7)	C711—C712—C713—C714	7(4)
C312—C313—C314—C39	2.3 (12)	C712—C713—C714—C79	−2(4)
C310—C39—C314—C313	−4.9 (12)	N73—C79—C714—C713	179 (2)
N33—C39—C314—C313	176.9 (7)	C710—C79—C714—C713	−2(3)
O41—N42—N43—C49	−0.1 (12)	O81—N82—N83—C89	−3(4)
C46—N42—N43—C49	−177.8 (6)	C86—N82—N83—C89	176 (2)
C41—N41—C43—C44	179.8 (7)	C82—N81—C83—C88	−173 (3)
C42—N41—C43—C44	−2.8 (12)	C81—N81—C83—C88	2(3)
C41—N41—C43—C48	−5.8 (12)	C82—N81—C83—C84	6(3)
C42—N41—C43—C48	171.7 (7)	C81—N81—C83—C84	−178 (2)
N41—C43—C44—C45	179.0 (7)	C88—C83—C84—C85	0(4)
C48—C43—C44—C45	4.4 (11)	N81—C83—C84—C85	−179.3 (19)
C43—C44—C45—C46	−6.2 (12)	C83—C84—C85—C86	0(4)
C44—C45—C46—C47	6.5 (12)	C84—C85—C86—C87	1(4)
C44—C45—C46—N42	−175.4 (7)	C84—C85—C86—N82	177 (2)
N43—N42—C46—C47	−3.4 (11)	N83—N82—C86—C85	−179 (3)
O41—N42—C46—C47	178.6 (7)	O81—N82—C86—C85	0(3)
N43—N42—C46—C45	178.7 (7)	N83—N82—C86—C87	−2(3)
O41—N42—C46—C45	0.7 (10)	O81—N82—C86—C87	177 (2)
C45—C46—C47—C48	−5.3 (12)	C85—C86—C87—C88	−2(4)
N42—C46—C47—C48	176.9 (7)	N82—C86—C87—C88	−179 (2)
C46—C47—C48—C43	3.3 (12)	C84—C83—C88—C87	−2(4)
N41—C43—C48—C47	−177.3 (7)	N81—C83—C88—C87	178 (2)
C44—C43—C48—C47	−2.8 (11)	C86—C87—C88—C83	3(4)
N42—N43—C49—C410	−4.1 (13)	N82—N83—C89—C814	−179 (3)
N42—N43—C49—C414	−180.0 (7)	N82—N83—C89—C810	8(4)
C414—C49—C410—C411	−3.6 (12)	C814—C89—C810—C811	7(4)
N43—C49—C410—C411	−179.2 (8)	N83—C89—C810—C811	179 (2)
C49—C410—C411—C412	6.0 (11)	C810—C89—C814—C813	−11 (4)
C416—N44—C412—C413	176.9 (7)	N83—C89—C814—C813	176 (2)

### Hydrogen-bond geometry (Å, °)

**Table:**

*D*—H···*A*	*D*—H	H···*A*	*D*···*A*	*D*—H···*A*
C15—H15···O11	0.95	2.35	2.708 (8)	101
C25—H25···O21	0.95	2.41	2.745 (8)	101
C35—H35···O31	0.95	2.32	2.651 (12)	100
C45—H45···O41	0.95	2.36	2.687 (12)	100
C55—H55···O51	0.95	2.38	2.722 (12)	100
C65—H65···O61	0.95	2.33	2.663 (16)	100
C110—H110···O11	0.95	2.15	2.742 (8)	119
C114—H114···O61^i^	0.95	2.51	3.328 (12)	144
C210—H210···O21	0.95	2.13	2.727 (10)	120
C214—H214···O31^ii^	0.95	2.50	3.402 (10)	158
C310—H310···O31	0.95	2.21	2.750 (12)	115
C410—H410···O41	0.95	2.17	2.732 (11)	117
C414—H414···O51	0.95	2.57	3.448 (12)	153
C510—H510···O51	0.95	2.09	2.684 (11)	120
C514—H514···O41^i^	0.95	2.48	3.323 (11)	148
C610—H610···O61	0.95	2.20	2.726 (16)	115
C614—H614···O11	0.95	2.58	3.471 (12)	156

Symmetry codes: (i) *x*−1, *y*, *z*; (ii) *x*+1, *y*, *z*.
